# Experimental evidence of high pressure decoupling between charge transport and structural dynamics in a protic ionic glass-former

**DOI:** 10.1038/s41598-017-07136-5

**Published:** 2017-08-01

**Authors:** Z. Wojnarowska, M. Rams-Baron, J. Knapik-Kowalczuk, A. Połatyńska, M. Pochylski, J. Gapinski, A. Patkowski, P. Wlodarczyk, M. Paluch

**Affiliations:** 1 Institute of Physics, University of Silesia, Uniwersytecka 4, 40-007 Katowice, Poland; 2 Silesian Center for Education and Interdisciplinary Research, 75 Pulku Piechoty 1A, 41-500 Chorzow, Poland; 3 Faculty of Physics, A. Mickiewicz University, Umultowska 85, 61-614 Poznan, Poland; 4 NanoBioMedical Centre, A. Mickiewicz University, Umultowska 85, 61-614 Poznan, Poland; 5 Institute of Non-Ferrous Metals, Sowinskiego 5, 44-100 Gliwice, Poland

## Abstract

In this paper the relaxation dynamics of ionic glass-former acebutolol hydrochloride (ACB-HCl) is studied as a function of temperature and pressure by using dynamic light scattering and broadband dielectric spectroscopy. These unique experimental data provide the first direct evidence that the decoupling between the charge transport and structural relaxation exists in proton conductors over a wide *T-P* thermodynamic space, with the time scale of structural relaxation being constant at the liquid-glass transition (τ_α_ = 1000 s). We demonstrate that the enhanced proton transport, being a combination of intermolecular H^+^ hopping between cation and anion as well as tautomerization process within amide moiety of ACB molecule, results in a breakdown of the Stokes-Einstein relation at ambient and elevated pressure with the fractional exponent *k* being pressure dependent. The d*T*_*g*_/d*P* coefficient, stretching exponent β_KWW_ and dynamic modulus *E*_*a*_/*ΔV* ^*#*^ were found to be the same regardless of the relaxation processes studied. This is in contrast to the apparent activation volume parameter that is different when charge transport and structural dynamics are considered. These experimental results together with theoretical considerations create new ideas to design efficient proton conductors for potential electrochemical applications.

## Introduction

The literature of the past two decades has experienced a comet-like boost of papers concerning ion conducting liquids and solids in general. Such huge academic and industrial interest in these materials is attributed to their unique properties that are highly relevant to a wide area of potential applications^1–3^. Among others, the most commonly explored research direction is to design efficient proton conductive systems for fuel cells and redox flow batteries^4, 5^. Following this strategy especially important became the family of protic ionic liquids and solids characterized by long-term thermal stability and high electric conductivity at low relative humidity^6^.

As our knowledge has advanced, it has been found that an extensive proton hopping through the ionic glass-former can be easily revealed by simple conductivity measurements^7–9^. Namely, at σ_dc_ higher than 10^−15^ Scm^−1^ (or conductivity relaxation times τ_σ_ shorter than 10^3^ s^10^) the σ_dc_(*T*) dependence shows a well-defined kink at the point of transition from non-Arrhenius to Arrhenius-like behavior which can be attributed to the liquid-glass transition of a given proton-conducting system. It has been well recognized that the origin for such phenomenon lies in the time scale separation between charge diffusion, realized by exceptionally fast proton migration, and relatively slow structural dynamics that remains in the range of 100–1000 s at *T*_*g*_^11, 12^. Additionally, very recently, it has also been demonstrated that for low-molecular protic ionic systems the value of σ_dc_ at *T*_*g*_ continuously increases with elevating pressure. Based on this it has been postulated that the proton transport becomes much more efficient in the compressed material^11, 13^. As a consequence, by squeezing the sample one can transform poor ionic liquids into superionic type, which creates new possibilities for potential fuel cell applications. Nevertheless, to verify this hypothesis and hence fully understand the mechanism of proton transport in condensed materials, the high pressure measurements of structural dynamics in protic ionic systems are required. Since this kind of experiment is technically complicated, especially close to the liquid-glass transition region, an analytical method based on the temperature-volume version of the Avramov entropic model^14^ and on the thermodynamic scaling concept^15^ was employed in the past to determine the pressure dependence of viscosity of protic ionic systems^13^. Based on these predictions it has been suggested that the crossover from Vogel-Fucher-Tamman (VFT) to Arrhenius-like behavior occurs at isostructural relaxation time or constant viscosity^13, 16, 17^. This means that compression is expected to enhance the decoupling between charge transport and structural dynamics of protic ionic glass-formers at the glass transition pressure P_g_. Nevertheless, this postulate has not been experimentally verified so far.

**Figure 1:**
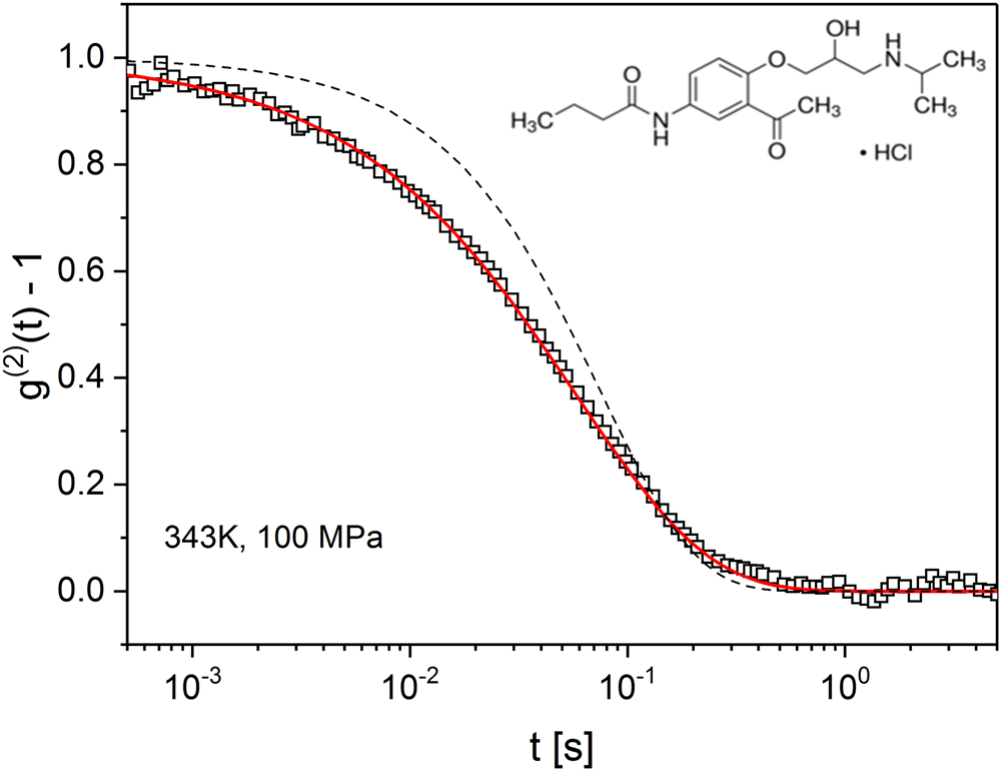
A typical normalized DLS time correlation function measured for ACB-HCl at 343 K and 100 MPa (points). The red solid line represents the KWW fit to the data. The dashed black line shows a simple exponential function to visualize the broadening of relaxation times distribution.

In this paper we examine the charge transport and structural relaxation of ionic glass-former obtained by proton transfer reaction between hydrochloride acid and acebutolol base (the chemical structure is presented in the inset to Fig. 1). The unique feature of this material are the chemical moieties actively involved in internal proton transfer. As a consequence efficient proton conductivity is expected in this system. The results of dynamic light scattering–photon correlation spectroscopy (DLS) experiments combined with broadband dielectric spectroscopy (BDS) measurements performed both at similar temperature and pressure conditions indicate that the charge transport is markedly faster than the structural dynamics of acebutolol HCl. Additionally, the combination of high pressure BDS and DLS data provide the first experimental evidence that the break of τ_σ_ (*T,P*) dependence, observed for a number of proton conducting systems, corresponds to isochronal structural relaxation times in the whole examined thermodynamic space. This, in turn, allows complex experimental examination of relaxation dynamics of protic ionic conductors at elevated pressure performed in terms of fractional Stokes-Einstein rule, dynamic modulus (*M*) and apparent activation volume ∆V^#^ and thus understanding the proton transport mechanism in ionic glass-formers at the microscopic level.

## Results and Discussion

### Are the charge transport and structural dynamics coupled in acebutolol hydrochloride (ACB-HCl)?

**Figure 2:**
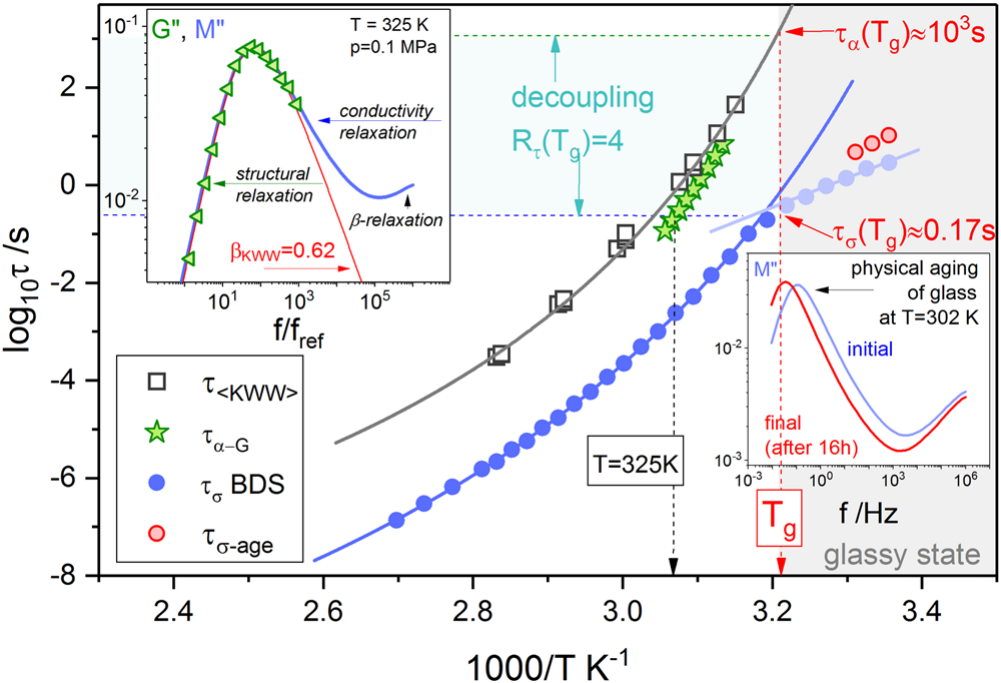
Relaxation map of ACB-HCl constructed using the data measured at ambient pressure conditions. The upper inset present the comparison of *M*″(*f*) and *G*″(*f*) spectra collected at 325 K. In the lower inset the evolution of conductivity relaxation spectra during physical aging at 302 K is presented.

Dynamic light scattering (DLS) measurements of ACB-HCl were performed both at ambient and elevated pressure (up to 160 MPa) (see Supplemental Materials for more details and refs 18–21). The representative autocorrelation function measured at 343 K and 100 MPa parameterized by means of the Kohlrausch-Williams-Watts (KWW) formula:

1 $${g}^{(1)}(\tau )=a+B\,\exp [-{(\frac{\tau }{{\tau }_{KWW}})}^{{\beta }_{KWW}}]$$

is presented in Fig. 1. Then, using the fitting parameters, the average KWW relaxation time 〈τ_KWW_〉 was calculated:

2 $$\langle {\tau }_{KWW}\rangle =\frac{{\tau }_{KWW}}{{\beta }_{KWW}}{\rm{\Gamma }}(\frac{1}{{\beta }_{KWW}})$$

where Γ(1/β_KWW_) is the Gamma function. The temperature evolution of average KWW relaxation times 〈τ_KWW_〉 obtained at ambient pressure conditions is presented in semilogarithmic scale in Fig. 2. As can be seen, the log〈τ_KWW_〉(*T* ^*−1*^) dependence is notably non-Arrhenius and remains above (longer times) the mechanical relaxation times τ_α-G_ determined directly from the intersection of storage *G*″(*f*) and loss *G’*(*f*) moduli, as usually for molecular liquids and polymers^22, 23^.

Generally, the structural relaxation times 〈τ_KWW_〉 of ACB-HCl rise from approximately 10^−4^s at high temperature to the value of 10^3^ s at the calorimetric glass transition point (*T*_*g*_^TMDSC^ = 315 K) and satisfactorily obey the Vogel-Fucher-Tamman (VFT) law, which are typical features of glass-forming systems^24^. At the same time, the logτ_σ_(*T* ^−*1*^) curve, with conductivity relaxation times determined directly from the maximum of the electric modulus spectra *M*″(*f*) (τ_σ _=1/2π *f*_*max*_), shows a clear kink reflecting the change from VFT-like to Arrhenius type behavior and at *T*_*g*_ reaches significantly lower value (around 0.17 s). This confirms a significant time scale separation between the conductivity and structural relaxation in the vicinity of the glass transition and indicates that the charge diffusion is still continued while the rotational motions of the ACB molecules are inhibited. Moreover, because a non-equilibrium glass is obtained, τ_σ_ is expected to increase during physical aging (τ_σ-age_)^25, 26^. This has been proved by the progressive shift of *M*″(*f*) peak towards lower frequencies during annealing at 302 K (see lower inset to Fig. 2). To quantify the decoupling of ion diffusion from the structural dynamics in ACB-HCl we have followed Moynihan *et al*.^27^ and calculated a decoupling index *R*_*τ*_(*T*_*g*_) = log[τ_α_(*T*_*g*_)/τ_σ_(*T*_*g*_)]. The value of 4 obtained from our data is much higher than *R*_*τ*_ determined for other hydrochloride salts^11^.

### Is the time scale separation between τ_α_ and τ_σ_ for ACB-HCl maintained under conditions of high compression?

**Figure 3:**
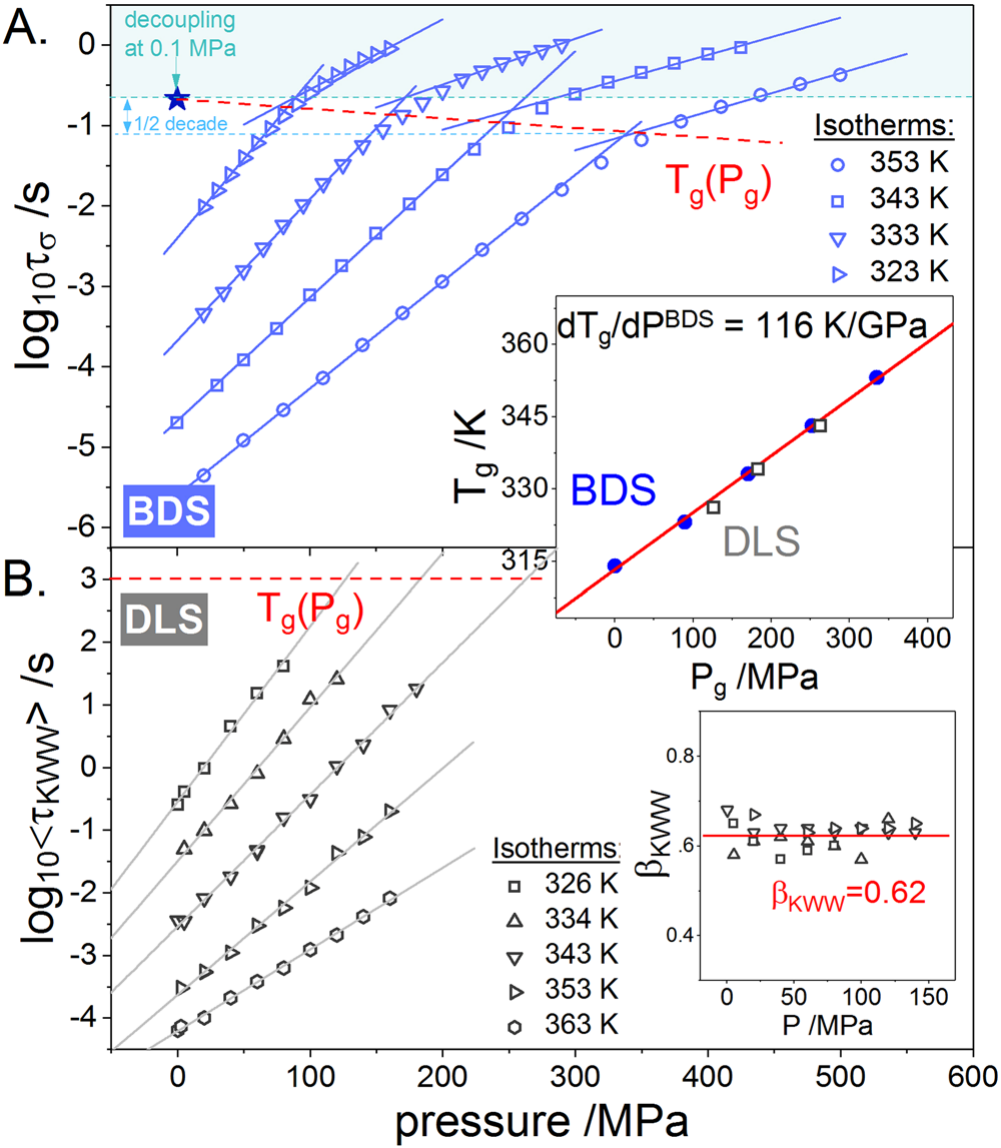
Isothermal measurements of conductivity (**A**) and structural (**B**) relaxation times of ACB-HCl. The insets present the pressure dependence of the *T*_*g*_ temperature (upper panel) and of the β_KWW_ parameter (lower panel).

To provide an answer to this issue first we have performed the high pressure conductivity measurements. The experimental details are described in Supplemental Material. As illustrated in Fig. 3A, the conductivity relaxation data of ACB-HCl recorded over a wide pressure range and at various isothermal conditions reveal a linear behavior well parameterized by means of the activation volume:

3 $$\mathrm{log}\,{\tau }_{\sigma }(P)=\,\mathrm{log}\,{\tau }_{{\sigma }_{0}}+\,\mathrm{log}\,e\frac{P{\rm{\Delta }}{V}^{\#}}{RT}$$

where log*τ*_*σ0*_ is the value of conductivity relaxation time at ambient pressure, *R* is the universal gas constant and Δ*V* ^*#*^ is an apparent activation volume commonly related to the local volume expansion required for ionic transport^28^. Nevertheless, a key feature of Fig. 3A is a clear kink of each τ_σ_(*P*) curve occurring around 0.1 s, indicating the glass transition pressure *P*_*g*_ of examined compound and thereby suggesting the time scale separation between structural and ionic displacement. However, to provide a strong experimental evidence of the decoupling phenomenon one needs the high pressure structural relaxation data of ACB-HCl. Herein, it is important to note that the dielectric loss spectra of ionic materials are entirely dominated by the dc-conductivity contribution. Hence, the information about structural dynamics of a given sample cannot be supplied by the BDS technique^24^. In this paper we took advantage of dynamic light scattering measurements to resolve this issue. The autocorrelation functions of ACB-HCl were recorded at five different temperatures and in the pressure range 0.1–160 MPa. Interestingly, the stretching parameter β_KWW_ obtained from fitting procedure of dynamic light scattering data is practically pressure independent (see inset to Fig. 3B). Additionally, it remains in good agreement with the Kohlrausch coefficient of 0.62 describing the shear *G*″(*f*) and electric modulus *M*″(*f*) spectra obtained at the same thermodynamic conditions (*T* = *T*_*g*_ + 10 K, *P* = 0.1 MPa) from mechanical and dielectric relaxation measurements (see inset to Fig. 2). As a consequence, the responses of supercooled liquid state of ACB-HCl measured via three different techniques display some specific features, reflected in approximately constant value of β_KWW_ parameter (β_KWW_ ≈ 0.62).

The pressure evolution of the DLS average relaxation times determined for ACB-HCl is presented in Fig. 3B. From this plot it can be easily verified that log〈τ_KWW_〉 data raise linearly in the whole available pressure range, also in the range of τ = 0.3 to 1 s, where _τσ_(*P*) data reveal the characteristic kink. Therefore, in analogy to ambient pressure conditions, the glass transition pressure can be determined from an isochronal definition, i.e. *P*_*g*_ = *P*(〈τ_KWW_〉≈10^3^ s). As illustrated in the inset panel to Fig. 3A, *T*_*g*_(*P*_*g*_) dependence assessed from the high pressure DLS experiments perfectly corresponds to values of *T*_*g*_ and *P*_*g*_ determined directly from the crossover bending points on the isothermal _τσ_(*P*) plot. These results provide the first experimental evidence that the inflection point of conductivity relaxation times measured both at ambient and elevated pressure always occurs at isochronal structural relaxation time equal to 1000 s. Having these data one can easily estimate the d*T*_*g*_/d_*P*_|_*P* = 0.1 MPa_ coefficient that reflects the pressure sensitivity of the glass transition temperature for the examined ACB-HCl. It was found to be equal to 0.116 K/MPa that is much lower than the value of d*T*_*g*_/d*P* reported in the past for other protic ionic conductors^8, 16, 29^.

### What is the origin of the time scale separation between the charge transport and structural dynamics occurring in ACB-HCl?

**Figure 4:**
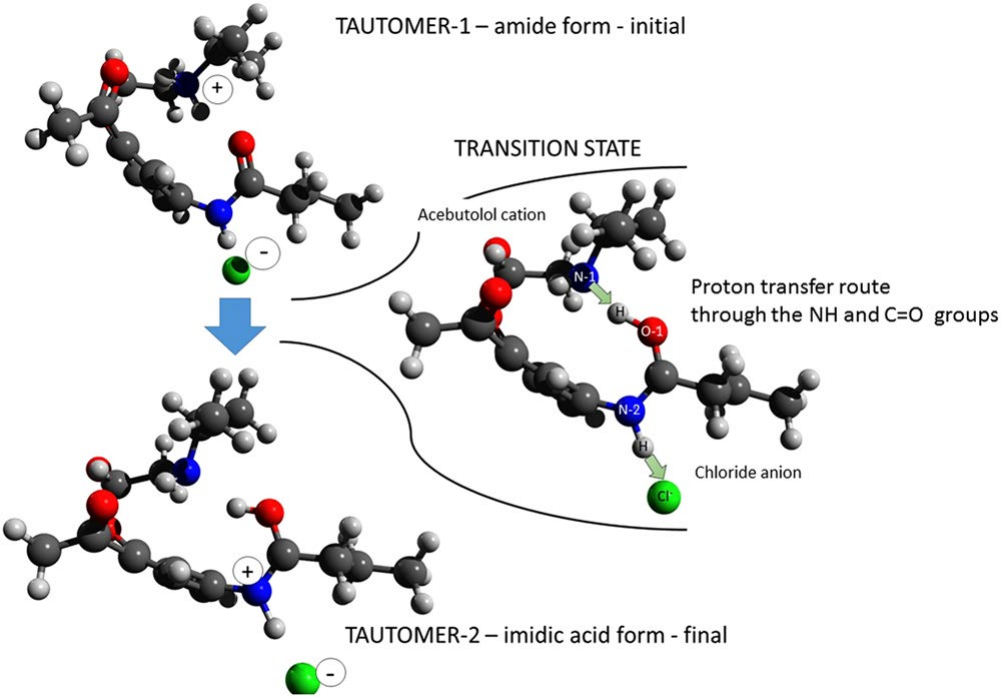
Proton transport mechanism in ACB-HCl.

It has been well established that regardless of T-P thermodynamic conditions the vehicle conduction is completely controlled by viscosity and therefore ionic transport is fully coupled to structural relaxation. Such picture is observed when the ionization of given protic systems is almost completed or when one counterion blocks the proton transfer^3, 4, 8^. On the other hand, conductivity relaxation was found to be faster than the structural dynamics if the proton hopping is involved in the charge transport^2^. Furthermore, it was postulated that under conditions of high compression the decoupling between τ_σ_ and τ_α_ becomes even more pronounced for proton conductors^8, 13^. This is because squeezing of a liquid not only reduces interatomic distances but also strongly affects H-bonds, the highways for efficient proton transport^30^. Having this in mind and looking at Fig. 3A, where the application of pressure brings the crossover of isothermal τ_σ_(*P*) curve toward shorter relaxation times, one can expect that H^+^ migration gives contribution to dc-conductivity of ACB-HCl. To provide more details on the charge transport in ACB-HCl we have performed DFT calculations (for more details see Supplemental Material). Due to the amide group in the chemical structure of ACB there is a possibility of intramolecular proton migration. In order to study this phenomenon, the proton transfer from nitrogen (N-1) to the oxygen of carboxyl group (O-1) in the vicinity of chloride anion was investigated. The activation energy of this process calculated on the B3LYP/6-311 G** level of theory corrected by the ZPE energy is equal to 15.9 kJ/mol and 1.2 kJ/mol for the reversed reaction. The reaction has only one stage, proton migration from N-1 atom, where the positive charge is localized, into oxygen in carbonyl group (O-1). Simultaneously, hydrogen from the N-2 is being pulled by the anion. In our case it is a chloride anion. Therefore, positive charge is transferred continuously from the nitrogen N-1, through the oxygen O-1, nitrogen N-2 and then it can jump to another electrically neutral acebutolol molecule through the chloride ion. As a result, initial acebutolol cation becomes neutral, while the neighboring neutral acebutolol molecule becomes ionized. At the same time, negatively charged chloride ion stays unchanged during the proton migration but it allows passing the proton from one acebutolol molecule to another within the so-called Grotthuss mechanism (see Fig. 4). These theoretical considerations are in agreement with recent reports indicating tautomerization process as an important factor accelerating fast proton hopping^31^. Thus, one can conclude that the efficient proton transport is the origin for decoupling phenomenon observed in ACB-HCl. Nevertheless, despite the relatively large time scale separation between structural dynamics and conductivity relaxation taking place at ambient conditions, decoupling does not change much with increasing pressure (around 0.5 decade), that is quantified by small value of d*R*_*τ*_/d*P* coefficient defined as $d\,\mathrm{log}\,{R}_{\tau }/dP=-d\,\mathrm{log}\,{\tau }_{cross}/dP$ (1.27 GPa^−1^). This result indicates that the H-bonded network created between counterions does not undergo any significant modifications up to 350 MPa and therefore the efficiency of proton conduction does not raise substantially. Thus, one can believe that also the molecular packing of acebutolol molecules is poorly affected by squeezing. This assumption corresponds well with weak pressure sensitivity of the glass transition temperature that is reflected in the low value of d*T*_*g*_/d*P* coefficient found for ACB-HCl. Interestingly, in the group of protic ionic conductors ACB-HCl is the first compound characterized by the lowest values of both d*T*_*g*_/d*P* and d*R*_*τ*_/d*P* parameters. At the highest extreme is carvedilol dihydrogen phosphate with d*T*_*g*_/d*P* = 0.170 K/MPa and d*R*_*τ*_/d*P* = 7.9 GPa^−1^ ^11^. On the other hand, one can recall aprotic prototypical ionic conductor CKN, with d*T*_*g*_/d*P* = 0.06 K/MPa and decoupling index practically independent of *T-P* conditions^32^.

### Are the activation volume parameters (Δ*V*^#^) determined from conductivity and structural relaxation the same for the examined hydrochloride salt?

Activation volume, defined as ${\rm{\Delta }}{V}^{\#}=2.303RT(d\,\mathrm{log}\,\tau /dP)$, where τ denotes either structural or conductivity relaxation time, is a very useful parameter to quantify the effect of pressure on the relaxation dynamics in ionic and nonionic glass-formers^33^. It is well established that ΔV^#^ parameter reflects the volume required for local motion of relaxing units and thus, it is directly related to the size of mobile species. So far the only available approximation of Δ*V* ^#^_α_ for ionic glass-formers came from analysis of d*T*_*g*_/d*P* coefficient and isobaric fragility *m*_*P*_ = d logτ_α_/d(*T*_*g*_/*T*)|_*P*=0.1MPa_ in terms of the following relation^28^:

4 $${\rm{\Delta }}{V}^{\#}=2.303R\cdot {\rm{d}}{T}_{g}/{\rm{d}}P\cdot {m}_{P}.$$

**Figure 5:**
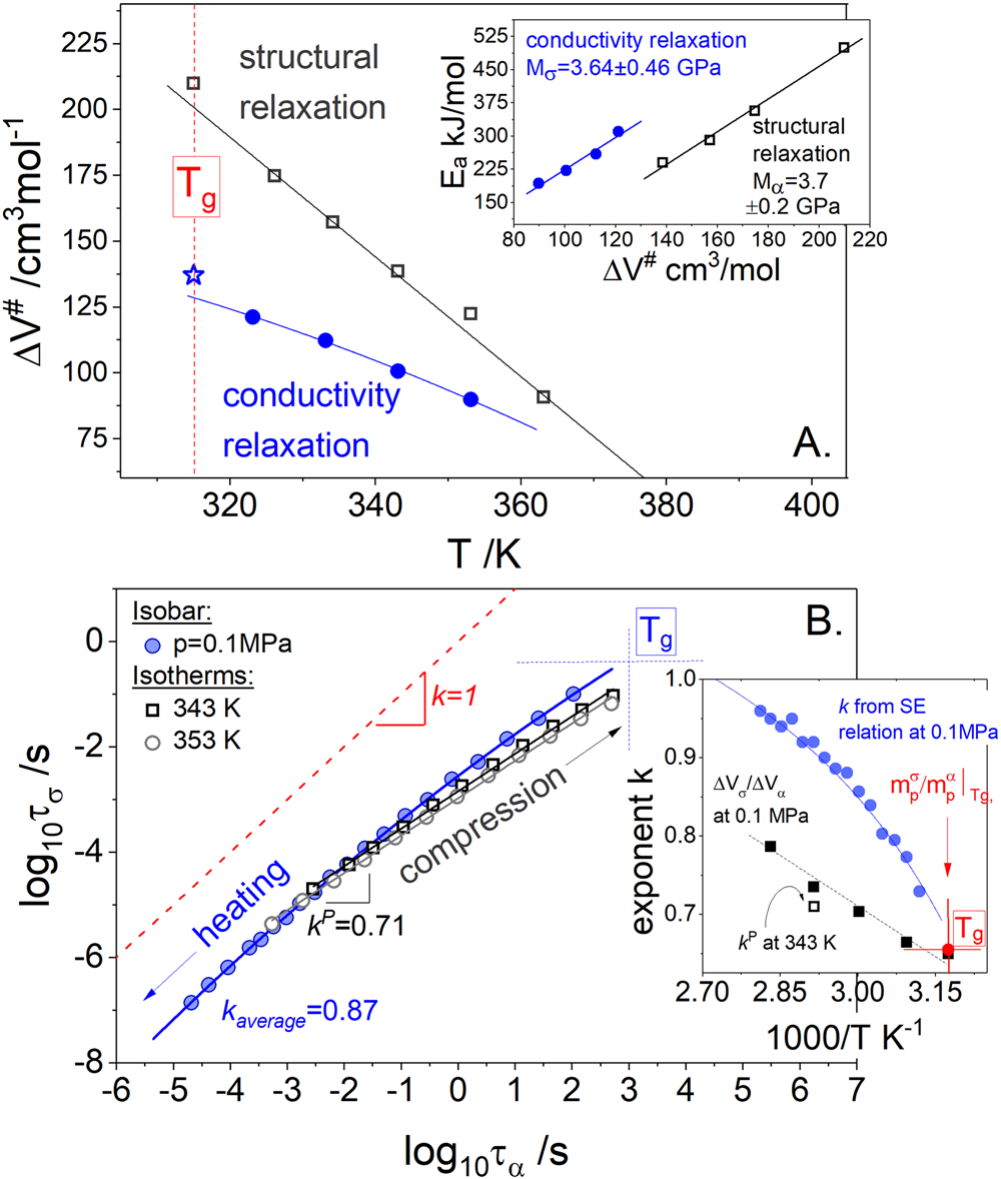
(**A**) Activation volume of structural relaxation and conductivity relaxation as a function of temperature. The Δ*V* ^*#*^ value at *T*_*g*_ was determined from Eq. 4 (stars). Inset panel presents the dynamic modulus calculated for structural and conductivity relaxation processes. (**B**): Test of the fSE at ambient and elevated pressures for ACB-HCl. Inset: the fractional exponent *k* as a function of reciprocal temperature compared to calculated *m*_*p*_^*σ*^ */m*_*p*_^*α*^ and Δ*V* ^*#*^_*σ*_/Δ*V* ^*#*^_*α*_ values.

To shed more light on this problem the values of the activation volume for ACB-HCl were determined directly from fitting of Eq. 3 to the BDS and DLS isothermal data. Note that this is the first time when relation between *ΔV* ^*#*^_*σ*_ and *ΔV* ^*#*^_*α*_ can be experimentally verified for protic ionic compound. Additionally, the obtained experimental values of Δ*V* ^*#*^_*σ*_ and Δ*V* ^*#*^_*α*_ were compared with those calculated from Eq. 4 using the value of fragility equals to 93 and 61 for log τ_α_(T^−1^) and log τ_σ_(T^−1^) data respectively. The results of such procedure, illustrated in Fig. 5A, reveal obvious differences and similarities between Δ*V* ^*#*^ parameter for structural relaxation and ion transport determined for the acebutolol salt. It appears that both Δ*V* ^*#*^_*σ*_ and Δ*V* ^*#*^_*α*_ calculated in the limit of ambient pressure decrease with increasing temperature that is a specific feature of the dynamics of supercooled liquids. However, when the same *T-P* conditions are considered the value of Δ*V* ^*#*^_*α*_ is much larger than Δ*V* ^*#*^_*σ*_. These results strongly support the idea that the mobile units governing the structural dynamics of the studied system are not the same as those responsible for conductivity relaxation. Taking into account the molar volume estimated for both acebutolol (that is within the range 220–280 cm^3^/mol) and perfectly spherical chloride anion (15 cm^3^/mol^34^), one can assume that the ACB^+^ and Cl^−^ give different contribution to ionic conductivity and structural relaxation. While acebutolol molecules dominate structural dynamics, the motions of smaller and more mobile chloride anions, supported by a contribution of the proton hopping mechanism, can be considered as a source of efficient charge transport. From Fig. 5A it is also evident that the values of Δ*V* ^*#*^_*α*_ and Δ*V* ^*#*^_*σ*_ markedly differ from each other near the liquid-glass transition and become almost the same at relatively high temperatures. This result is in agreement with general findings that the proton hopping conduction is progressively dominated by vehicle-type mechanism with increasing temperature. An additional outcome of Fig. 5A, is a good agreement between Δ*V* ^*#*^ obtained directly from experiments and those estimated at *T*_*g*_ using Eq. 4.

### Is the “dynamic modulus” the same for both charge transport and structural relaxation?

The most striking result is obtained from the joint study of the activation volume and activation energy ${E}_{a}=2.303R(d\,\mathrm{log}\,\tau /d{T}^{-1})$, performed in terms of the so-called “modulus of elasticity” (*M*) defined as the ratio of these two activation parameters^35^. The inset in Fig. 5A presenting the *E*_*a*_(Δ*V* ^*#*^ *)* dependence, enables a direct comparison to be made between dynamic modulus determined from *T-P* behavior of τ_σ_ and τ_α_, respectively (i.e. between *M*_*σ*_ and *M*_*α*_). The main significance of this plot is that the conductivity and structural relaxation data form two separate straight lines with approximately the same slope, i.e. similar value of the dynamic modulus. It means that regardless of the relaxation processes studied the changes in *E*_*a*_ are proportional to variations of Δ*V* ^*#*^ in ACB-HCl. On the other hand, the pronounced separation of these two lines along the horizontal axis indicates the decoupling of the ion motions from structural relaxation occurring in the examined system. This remains in good agreement with the observation made by Ingram *et al*.^36^ for inorganic ionic conductors.

### Is the Stokes-Einstein relation satisfied over a wide T-P thermodynamic range?

To provide a deeper understanding of the decoupling phenomenon in the examined protic salt it is worth to discuss the relationship between the ionic transport and structural relaxation in terms of the phenomenological fractional Stokes-Einstein (SE) relation τ_σ_·τ_α_^−*k*^  ≈ const. A number of theoretical and experimental studies performed in the past for ionic systems clearly indicate that the log τ_σ_ vs. log τ_α_ form a straight line with a slope close to unity when the ion transport is fully controlled by viscosity^37, 38^ and deviate from this general rule if these two processes are decoupled from each other^13^. Furthermore, it has been found that regardless of *T-P* conditions the SE relation is satisfied if the conductivity of ionic system is governed by classical vehicle conduction^29^. However, up to now there is no experimental evidence to support this hypothesis also for decoupled proton conductors. From this point of view it is of general significance to examine the SE criterion for ACB-HCl in the whole available temperature and pressure thermodynamic space. In this respect the values of τ_σ_ and τ_α_ measured at various *T-P* conditions have been plotted in Fig. 5B. From the first sight it is apparent that the log τ_σ_ = *f*(log τ_α_) data obtained for isothermal and isobaric pathways do not superimpose each other and markedly deviate from a linear behavior. This result indicates that decoupling exponent *k* is sensitive to temperature and pressure changes. As depicted in the inset in Fig. 5B, at 0.1 MPa exponent *k* decreases from 0.85 at relatively high temperatures to 0.65 in the vicinity of the liquid-glass transition. Moreover, when the isochronal conditions are considered (i.e. τ_σ_ = const or τ_α_ = const), SE exponent is always smaller under higher pressure. This is in agreement with the recent predictions made for a strongly decoupled system carvedilol dihydrogen phosphate showing that the value of *k* is significantly smaller at elevated pressure^13^. On the other hand, an extremely interesting result can be obtained by differentiating the logarithmic form of SE rule with respect to *T*_*g*_ */T* and combining the obtained formula with Eq. 4 and isobaric fragility *m*_*P*_. Such procedure leads to the straightforward relation that links the decoupling exponent *k* with the ratio of activation volumes and fragilities determined for both conductivity and structural relaxation:

5 $${\frac{{\rm{\Delta }}{V}_{\sigma }^{\#}}{{\rm{\Delta }}{V}_{\alpha }^{\#}}|}_{P=0.1MPa}={\frac{{m}_{P}^{\sigma }}{{m}_{P}^{\alpha }}|}_{P=0.1MPa}=k$$

As illustrated in the inset in Fig. 5B, *m*_*p*_^*σ*^/*m*_*p*_^*α*^ determined at *T*_*g*_, as well as Δ*V* ^*#*^_*σ*_/Δ*V* ^*#*^_*α*_ calculated at the same *T-P* conditions correspond well with the value of the Stokes-Einstein exponent. Nevertheless, when the supercooled liquid region is considered, Eq. 5 is no longer satisfied. This is because the isochronal definition of the glass transition temperature/pressure (τ_σ_(*T*_*g*_,*P*_*g*_) = const), being a fundamental assumption of Eq. 4, is not valid in strongly decoupled systems including ACB-HCl. Another confirmation of such statements comes from high pressure studies of carvedilol dihydrogen phosphate, for which the exponent *k* = 0.6 and the value of Δ*V* ^*#*^_*α*_(*T*_*g*_) was found to be more than five times larger than that of Δ*V* ^*#*^_*σ*_(*T*_*g*_).

## Conclusions

The examined protic ionic glass-former is the first example of hydrochloride salt revealing strong decoupling (*R*_*τ*_(*T*_*g*_) = 4) of ion motions and structural relaxation in the vicinity of the liquid-glass transition. According to DFT calculations the reason of such effective charge transport lies in combined intermolecular proton transport between cation and anion and intramolecular H^+^ hopping within amide moiety of ACB molecule. Thus, the example of ACB-HCl confirms that tautomerization process is a critical factor accelerating fast proton conductivity in ionic glass-formers. This knowledge offers new ideas to design an efficient proton conductors for various electrochemical applications.

Having acebutolol HCl as an example we have also confirmed experimentally that regardless of temperature and pressure thermodynamic conditions the characteristic crossover of temperature dependence of conductivity relaxation times from VFT-like to Arrhenius behavior (observed in many others protic ionic conductors and reflecting the existence of fast proton transport) occurs always at the same structural relaxation time (τ_α = _1000 s), i.e. under isochronal conditions. Surprisingly, the large time scale separation between charge transport and structural relaxation observed at ambient conditions is not accompanied by its significant changes of the decoupling index at elevated pressure. These results clearly indicate that the significant modifications of structural dynamics (reflected in large value of d*T*_*g*_/d*P* coefficient) are required to enhance the proton transfer under high pressure conditions.

## Electronic supplementary material

Supplementary Information
