# Supplementary material for: Experimental evidence of high pressure decoupling between charge transport and structural dynamics in a protic ionic glass-former
Source: Sci Rep. 2017 Aug 1;7:7084. doi: 10.1038/s41598-017-07136-5 (PMC5539233; doi:10.1038/s41598-017-07136-5)
Supplement: Supplementary file 1 — Supplementary Information [file 41598_2017_7136_MOESM1_ESM.pdf]

Supplemental material

Experimental evidence of high pressure decoupling between charge transport and structural dynamics in a protic ionic glass-former

Z. Wojnarowska[1,2], M. Rams-Baron[1,2], J. Knapik-Kowalczuk[1,2], A. Połatyńska[3], M. Pochylski[3], J. Gapinski[3,4], A. Patkowski[3,4], P. Wlodarczyk[5], M. Paluch[1,2]

[1]Institute of Physics, University of Silesia, Uniwersytecka 4, 40-007 Katowice, Poland
[2]Silesian Center for Education and Interdisciplinary Research, 75 Pulku Piechoty 1A, 41-500 Chorzow, Poland
[3] Faculty of Physics, A. Mickiewicz University, Umultowska 85, 61-614 Poznan, Poland
[4] NanoBioMedical Centre, A. Mickiewicz University, Umultowska 85, 61-614 Poznan, Poland
[5] Institute of Non-Ferrous Metals, Sowinskiego 5, 44-100 Gliwice, Poland

Experimental details

I Examined sample

The acebutolol hydrochloride (ACB-HCl) with the chemical name N-(3-Acetyl-4-(2-hydroxy-3 (isopropylamino)propoxy)phenyl)butyramide hydrochloride, M_W= 372.88 g/mol and the chemical structure presented in Fig.1S was supplied from Sigma Aldrich (CAS Number 34381-68-5) and it was used without any further purification. The starting material was completely crystalline with the melting point at 417 K determined by standard DSC technique. The examined sample is good glass-forming liquid with T_g = 320 K (rate 10K/min) and no tendency toward recrystallization during heating (see Fig. 1S).

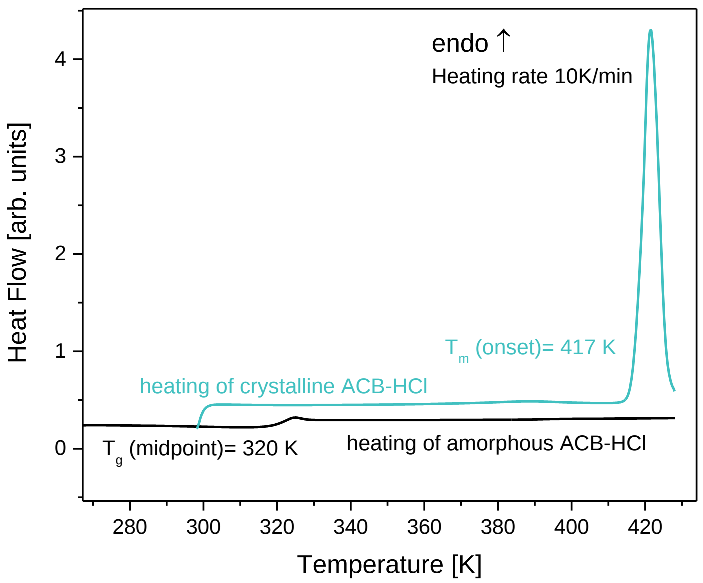

Fig. 1S Thermal analysis of crystalline and amorphous forms of ACB-HCl. All DSC thermograms were obtained during heating at a rate of 10 K/min. The inset shows the chemical structure of studied sample.

II Differential Scanning Calorimetry

Thermodynamic properties of ACE-HCl were examined by means of Mettler-Toledo DSC 1 STAR^e System. The measuring device was calibrated for enthalpy and temperature using zinc and indium standards. The instrument was equipped with HSS8 ceramic sensor having 120 thermocouples and liquid nitrogen cooling station. The samples were measured in an aluminum crucible (40 μL). Melting point was determined as the onset of the endothermic peak, while the glass transition temperature was determined as the midpoint of the heat capacity increment.

During the standard DSC experiment the crystalline sample was heated up from 300 K to 430 K, whereas the glassy ACE-HCl was heated up from 270 K to 430 K. Heating rate applied in both these cases was equal to 10 K/min.

To determine the frequency dependent calorimetric glass transition temperature, the stochastic temperature-modulated differential scanning calorimetry (TOPEM) method was employed. Mentioned measurement was performed in the temperature range from 283 K to 343 K with a heating rate of 0.5 K/min.

III Broadband Dielectric Spectroscopy

Isobaric dielectric measurements at ambient pressure from 10^{-1} to 10^7 Hz were carried out using a Novo-Control GMBH Alpha dielectric spectrometer. For the isobaric measurements, the sample was placed between two stainless steel electrodes of the capacitor with a gap of 0.1 mm. The dielectric spectra of acebutolol HCl were collected over a wide temperature range from 355 to 173 K. The temperature was controlled by the Novo-Control Quattro system, with the use of a nitrogen gas cryostat. Temperature stability of the samples was better than 0.1 K.

The physical aging experiments were performed at three different temperatures 298, 300 and 302 K. Each time the sample was heated above T_g for 15 minutes and then immediately cooled down in Alpha analyzer to desired annealing temperature.

For the pressure dependent dielectric measurements we used capacitor, filled with the ACB-HCl sample, which was next placed in the high-pressure chamber and compressed using the silicone oil. Note that during the measurement the sample was in contact with stainless steel and Teflon. Pressure was measured by the Nova Swiss tensometric pressure meter with a resolution of 0.1 MPa. The temperature was controlled within 0.1 K by means of a liquid flow provided by a Weiss fridge.

According to general practice, the dielectric experimental data of ACB-HCl were analyzed in modulus representation. As presented in the upper panel of Fig. 2S the imaginary part of complex electric modulus M"(f) form a well resolved peak with the maximum directly indicating conductivity relaxation times $\tau_\sigma=1/2\pi f_{max}$.

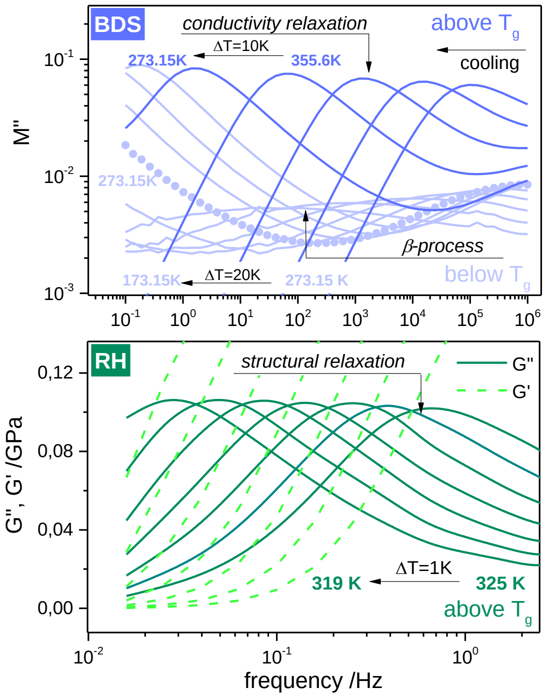

Fig. 2S Top panel: the frequency dependences of M" for ACB-HCl at the labelled temperatures (i.e. dielectric spectra). Bottom panel: the frequency dependences of G' and G" for ACB-HCl at the labelled temperatures (i.e. mechanical spectra).

IV Dynamic light Scattering

Dynamic light scattering setup consisted of a Coherent Genesis MX SLM laser (1W, 532 nm) operating at the power of 100 mW, a Perkin-Elmer SPCM-AQR avalanche photodiode, an ALV7002 digital correlator controlled by ALV software ver. 3.0 and high pressure cell. Depolarized component of the scattered light was delivered to the diode by a monomode optical fiber. A homemade high pressure cell [1,2] was pressurized with nitrogen using a NovaSwiss compressor model (554.2320-4). The samples were prepared by rapid melting of the powder in a home-made conical heater (2 cm diameter) with a hole in the center directing the drops of melted samples to a glass cell. The glass cells of 12 mm outer diameter were silanized inside to minimize the strain exerted on the walls by cooled sample. ALV correlator software ver. 3.0 was used to fit the measured correlation function with the KWW model function.

V Mechanical Spectroscopy

Dynamic mechanical measurements were performed by means of an ARES-G2 rheometer. The frequency dependencies of the storage (G′) and loss (G″) shear moduli were measured in the frequency range from 0.1 to 100 rad/s. The representative G'(f) and G"(f) spectra are depicted in the lower panel of Fig. 2S. Experiments were performed upon heating in the temperature range from 317 K to 403 K. At the temperatures from 317 K to 343 K the ΔT was equal to 1 K, while at temperatures 345 K – 403 K the step of ΔT = 2 K. During all

measurement the sample was placed between aluminum parallel plates with the gap equal to 0.5 mm and the plate diameters equal to: (i) 8 mm at temperatures from 317 K to 357 K, and (ii) 25 mm at temperatures from 357 K to 403 K. Shear deformation was applied under conditions of controlled strain and linear viscoelastic response.

VI DFT calculations

All the calculations were performed in the orca 3.0.3 program [3] within the framework of density functional theory. For this method, hybrid density functional B3LYP was chosen as it is universal for geometry optimizations, vibrational spectra calculations and energy evaluations. Gaussian triple zeta type basis set 6-311G** [4] with additional polarization functions was used for all the levels of calculations [5,6]. Transition state theory was used for evaluation of proton transfer reaction. In order to obtain geometry of transition state, eigenmode following method was used as it is implemented in orca 3.0.3. Energy differences between transition state and minima are the energy barriers for reaction. The energy barrier was evaluated as a sum of electronic and vibrational energy (zero point energy correction). In all calculations empirical D3 corrections were used to properly describe dispersion interactions.

---

[1] Fytas, G., Patkowski, A., Meier, G. & Dorfmüller, Th. Separation of two relaxation processes in bulk polymers using photon correlation spectroscopy at high pressures. *Macromolecules* **15**, 214 (1982).

[2] Fytas, G., Patkowski, A., Meier, G. & Dorfmüller, Th. Pressure- and temperature-dependent photon correlation study of bulk poly(ethyl acrylate) above the glass transition temperature. *Macromolecules* **15**, 870 (1982).

[3] Neese, F. (2012) The ORCA program system, Wiley Interdiscip. Rev.: Comput. Mol. Sci., 2, 73-78

[4] Krishnan, R., Binkley, J.S., Seeger, R. & Pople, J.A. Self-consistent molecular orbital methods. XX. A basis set for correlated wave functions *J. Chem. Phys*. 72, 650 (1980).

[5] Grimme, S., Ehrlich, S. & Goerigk, L. Effect of the damping function in dispersion corrected density functional theory *J. Comput. Chem*. (2011), 32, 1456-1465

[6] Grimme, S., Antony, J., Ehrlich, S. & Krieg, H. A consistent and accurate ab initio parametrization of density functional dispersion correction (DFT-D) for the 94 elements H-Pu *J. Chem. Phys*. 132, 154104 (2010).
